# Selective breast/gynecologic pathology fellowship training in the United States: Experience of program directors

**DOI:** 10.1016/j.acpath.2023.100103

**Published:** 2024-02-14

**Authors:** Mohamed Mokhtar Desouki, Ian S. Hagemann, Thaer Khoury, Oluwole Fadare, Rohit Bhargava, Jennifer L. Clark, Michael T. Deavers, Julie M. Jorns, Ashraf Khan, Ediz F. Cosar, Rouzan G. Karabakhtsian, Molly E. Klein, Andre Pinto, Muhammad Ali

**Affiliations:** a Department of Pathology, Roswell Park Comprehensive Cancer Center, Buffalo, NY, USA; b Department of Pathology and Anatomical Sciences, University at Buffalo, Buffalo General Medical Center, Buffalo, NY, USA; c Jacobs School of Medicine and Biomedical Sciences, State University of New York at Buffalo, USA; d Department of Pathology & Immunology, Washington University School of Medicine, St. Louis, MO, USA; e Department of Pathology, University of California San Diego School of Medicine, San Diego, CA, USA; f Department of Pathology, University of Pittsburgh Medical Center, Magee-Women's Hospital, Pittsburgh, PA, USA; g Department of Pathology, UMass Chan Medical School, Worcester, MA, USA; h Department of Pathology, Houston Methodist Hospital, Houston, TX, USA; i Department of Pathology, Medical College of Wisconsin, Milwaukee, WI, USA; j Department of Pathology, UMass Chan Medical School-Baystate, Baystate Health, Springfield, MA, USA; k Department of Pathology, Montefiore Medical Center, Albert Einstein College of Medicine, Bronx, NY, USA; l Department of Pathology, University of Minnesota, Indianapolis, MN, USA; m Department of Pathology, University of Miami Health System, Miami, FL, USA

**Keywords:** Breast, Gynecologic, Pathology, Fellowship training

## Abstract

Published data on combined breast and gynecologic [breast/gyn] surgical pathology fellowship training programs are limited. Our study aimed to survey the landscape of such fellowships in the United States (US), including specific information about their characteristics and the educational activities therein. Using web searches, we identified programs offering combined breast/gyn surgical pathology fellowship training. We developed a 26-item questionnaire asking program directors to report on the characteristics of their fellowship training structure. The search revealed 25 academic based programs offering one-year combined breast/gyn fellowship training, predominantly located (40 %) in the Northeast area. The following data was obtained: 44 % of the programs were accredited by the ACGME, 82 % required >19 weeks of breast and gyn service, and 69.6 % accepted the common application, 54.5 % of programs require completion of a research project for graduation. An annual average of 3000 breast and 3000 gyn cases appears to be the usual volume of cases. Interestingly, only 36 % of the program directors are graduates of a combined breast/gyn fellowship program. In conclusion, we present the most comprehensive and up-to-date census of combined breast/gyn pathology fellowships in the US. Our study provides valuable information on the current state of combined breast/gyn pathology fellowship training. The information will be helpful to current and prospective trainees, as well as program leaders.

## Introduction

Surgical pathology is a medical subspecialty focused on the study of tissue samples to help in diagnosing diseases, assist in formulating management plans, and study prognostic and predictive markers.^1^ The number of subspecialties of training in anatomic pathology has grown rapidly over the past decade, with more than 13 subspecialties now being recognized.^2^, ^3^, ^4^ Although there are general surgical pathology fellowships that cover a range of organ systems, most subspecialty fellowships (“selective pathology” programs) focus on a single organ system.

The high volume of breast and gyn specimens, complex surgical procedures, frequent updates and new classifications mandate up-to-date knowledge. Such refined skills for pathologists are best acquired through subspecialty training. In recent years, there have been remarkable advances in our understanding of the molecular basis of diseases, and the advent of next-generation molecular biology techniques have further contributed to the demand for experts and leaders in the field, driving the need for subspecialty fellowship programs.^5^ These factors have made breast and/or gyn pathology some of the most common pathology fellowship areas in the United States (US).^6^

There is, however, no central repository of data on fellowships in each area of surgical pathology. A database published by the Association of Pathology Chairs for the academic year 2025–2026 listed combined breast/gyn in addition to other subspecialties (Table 1).^2^^,^^4^

**Table 1:** List of selective surgical pathology fellowship training in the United States published by the Association of Pathology Chairs for the academic year 2025–2026.

Fellowship	Number of slots
General surgical or oncologic pathology	72
Gastrointestinal ( ± liver, pancreas)	56
Combined breast/gyn compared	23
Genitourinary (GU)	22
Gynecologic (gyn)	19
Breast	18
Renal	15
Head and neck±endocrine	13
Bone and soft tissue	11
Flexible subspecialty	8
Pulmonary	7
combined pulmonary and cardiovascular	5
combined GU + Renal	4
Cardiovascular	2
Transplant	1
combined clinical informatics + surgical pathology	1
GU + gyn	1

Our study aimed to determine the following characteristics of combined breast/gyn pathology fellowships in the US: Institution type, educational activities, and the structure of the pathology department. Additionally, we collected survey data on typical curriculum and educational goals during the fellowship training period. Describing current training practices may facilitate standardization of training across the US.

## Materials and methods

An online search was completed to identify programs that offer combined breast/gyn selective surgical pathology fellowship training. The search included the Association of Pathology Chairs website,^2^ the American Medical Association fellowship list,^3^ the Pathology Outlines website,^7^ the International Society of Gynecological Pathologists,^8^ and personal communications by the corresponding author (MMD).

A 26-item questionnaire on Microsoft 365 Forms to collect characteristics of the fellowship programs (Supplemental Material 1) was created. The link to the questionnaire was sent to all program directors of combined breast/gyn fellowships via e-mail in August 2022 with follow-up emails, allowing the programs to update their data or reminding them to respond. Survey responses and publicly available data were collated for analysis.

## Results

The search identified 25 programs (“all programs”) that currently offer one year training in combined breast/gyn fellowship training in the US (Table 2). Eleven of twenty-five (44 %) programs responded to the survey “responding/participating programs.” The first participating program reported that fellowship training started in 2003 (University of Pittsburgh Medical Center), and the most recent one started in July 2023 (Roswell Park Comprehensive Cancer Center).

**Table 2:** List of all combined breast/gyn fellowships in the US identified by the search strategy as of 2023.

#^a^	Institution	Slots	State	ACGME accredited
1	University of Alabama at Birmingham	1	AL	No
2	University of California Davis	1	CA	No
3	**University of California San Diego**	1	CA	No
4	University of Colorado	1	CO	No
5	**University of Miami/Jackson Health System**	2	FL	Yes
6	University of Chicago, NorthShore Program	1	IL	No
7	**Baystate Health - University of Massachusetts Chan Medical School**	1	MA	Yes
8	**Umass Chan Medical Center (Worcester)**	1	MA	Yes
9	Detroit Medical Center/Wayne State University School of Medicine	1	MI	Yes
10	**University of Minnesota**	1	MN	Yes
11	**Washington University**	2	MO	Yes
12	**University at Buffalo**	1	NY	Yes
13	**Montefiore Medical Center/Albert Einstein College of Medicine**	1	NY	Yes
14	Zucker School of Medicine at Hofstra/Northwell	1	NY	Yes
15	NYU Grossman School of Medicine	1	NY	No
16	University of Rochester	1	NY	No
17	Oregon Health & Science University	1	OR	No
18	**Magee-Womens Hospital/University of Pittsburg**	3	PA	No
19	Women and Infants Hospital of Rhode Island/Brown University	1	RI	No
20	**Houston Methodist Hospital**	1	TX	Yes
21	University of Virginia	1	VA	No
22	University of Utah		UT	No
23	University of Washington	2	WA	Yes
24	**Medical College of Wisconsin**	1	WI	No
25	University of Wisconsin School of Medicine and Public Health	1	WI	No

a The programs are sorted from A–Z according to the state where they are located. Programs in bold font are participating in the study (n = 11).

**Fig. 1:**
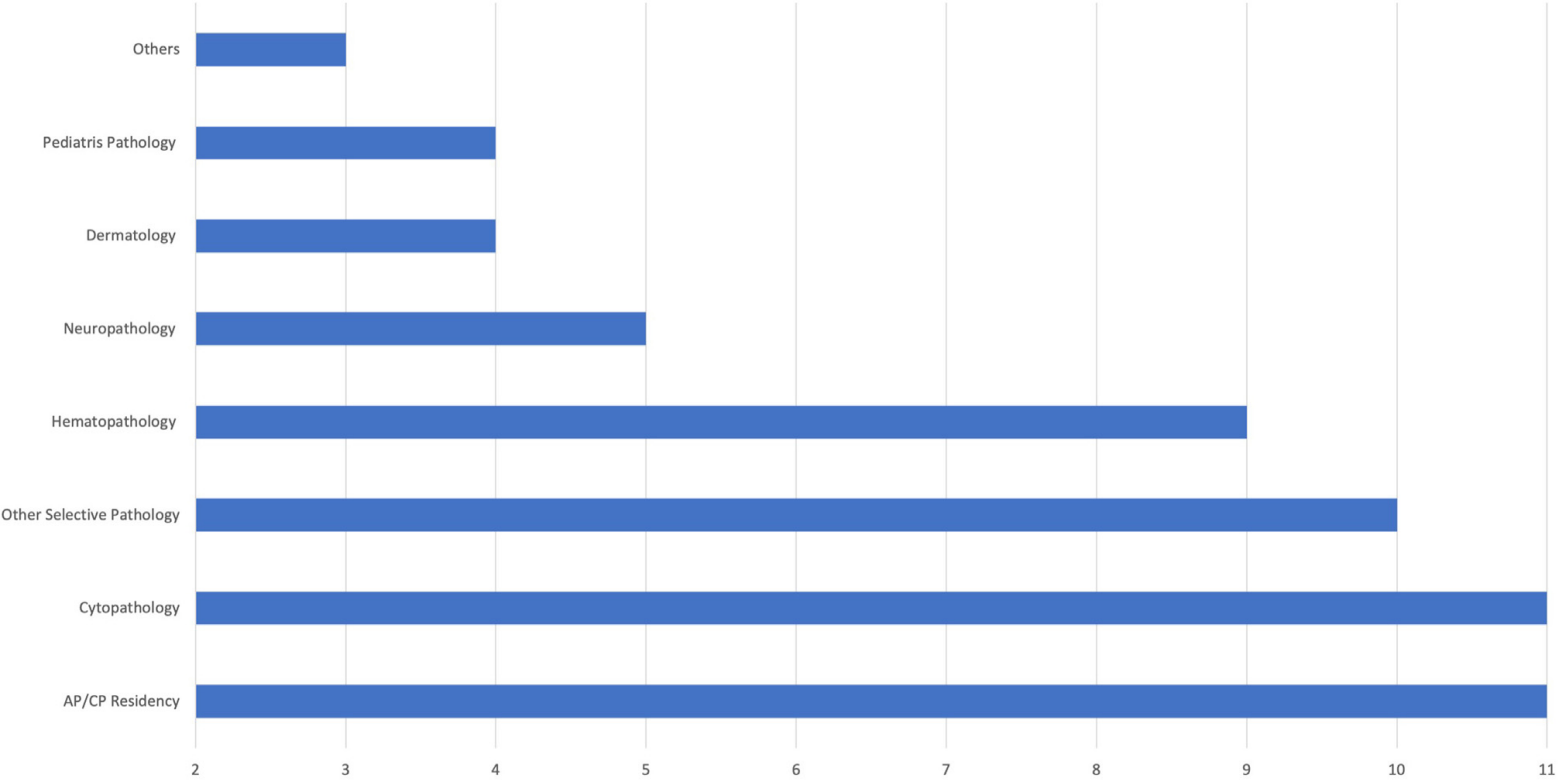
Fellowship training other than combined breast/gyn offered by the department of pathology of the participating programs.

**Fig. 2:**
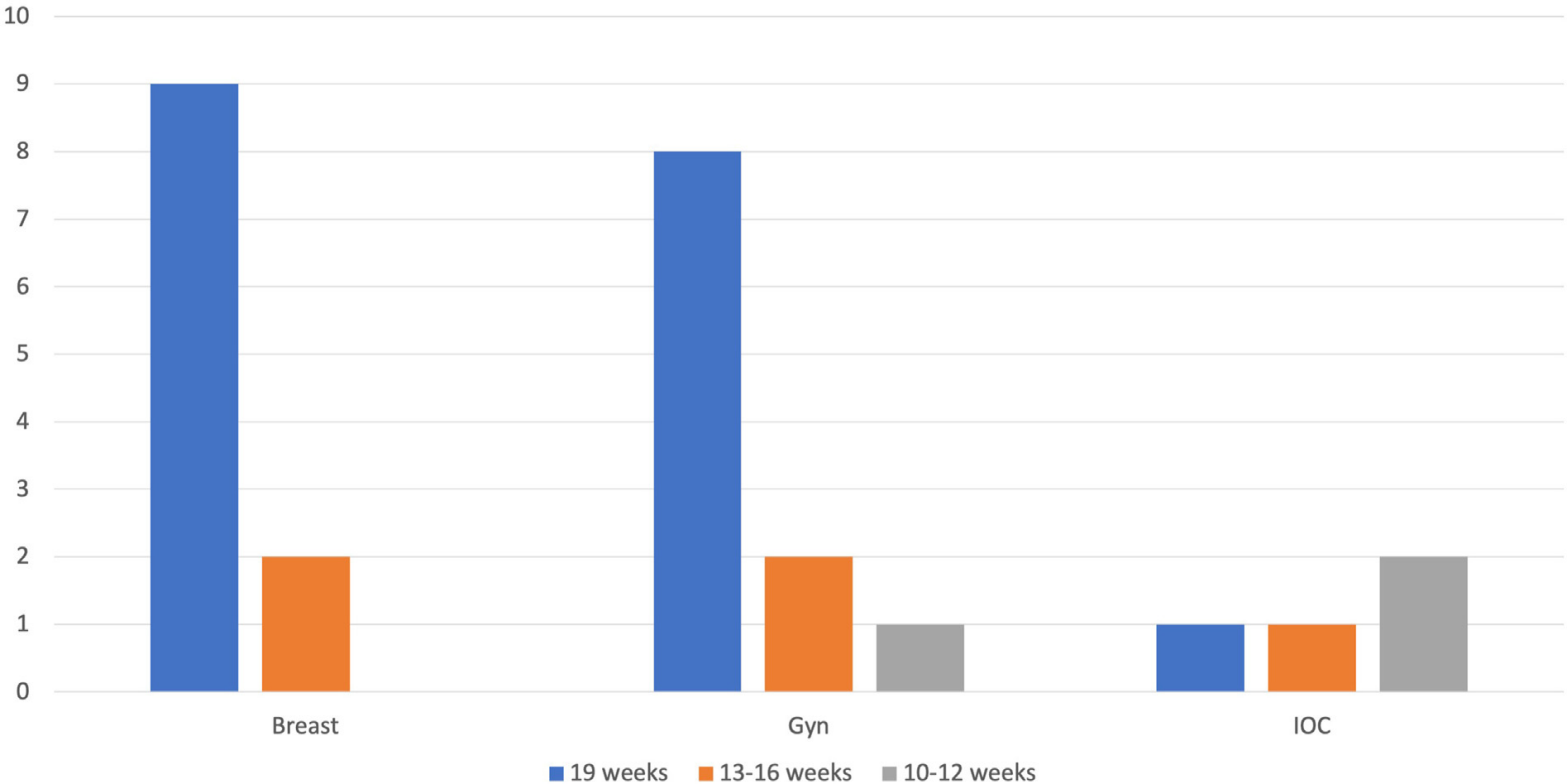
Rotations (in weeks) in breast, gyn, and intraoperative consultation (IOC) in participating programs.

**Fig. 3:**
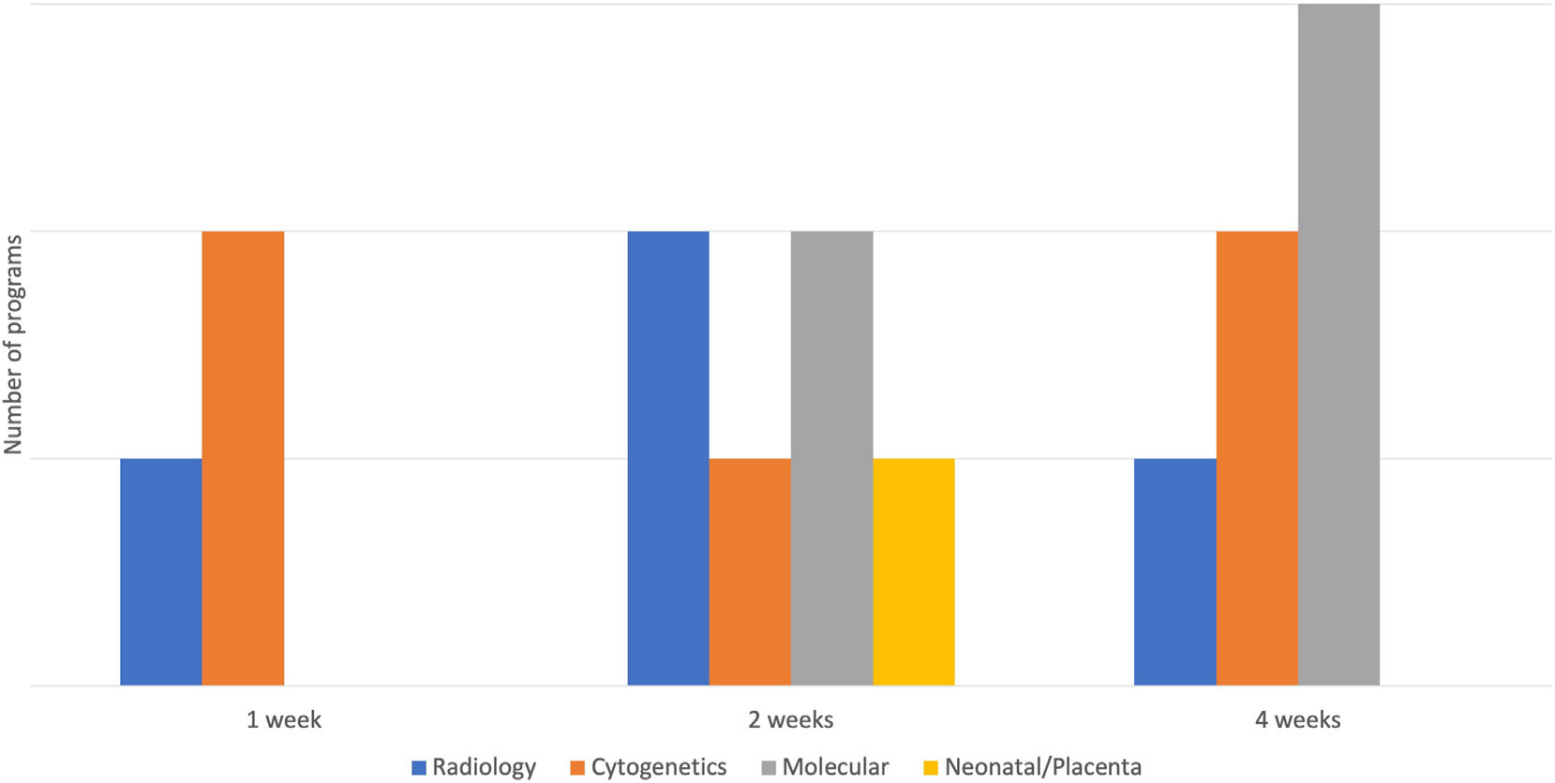
Additional rotations (in weeks) offered by participating programs.

All programs (25/25) are academics based in a university/medical school setting with an accredited pathology residency program. Table 2 lists all combined breast/gyn fellowships in the US identified by the search strategy as of 2023 along with the location and accreditation status by the Accreditation Council for Graduate Medical Education (ACGME). All the participating programs reported having additional (non-breast/gyn) fellowship programs in the department (Fig. 1). Responding programs vary in their block rotations in breast and gyn services as well as other rotations (Fig. 2, Fig. 3). Fellows participate in teaching medical students and residents in all participating programs.

**Fig. 4:**
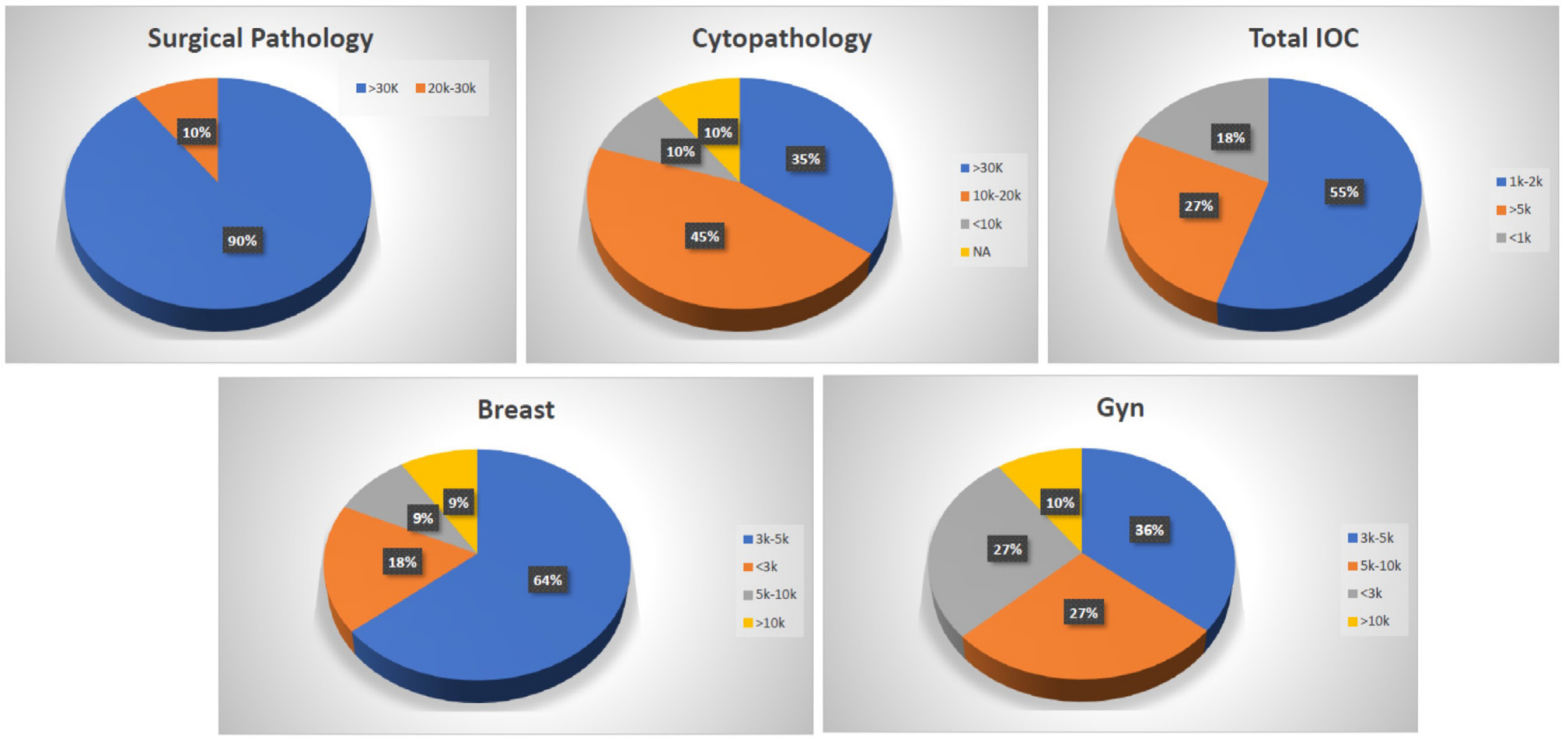
Average number of cases per year in the department of pathology of the participating programs.

**Fig. 5:**
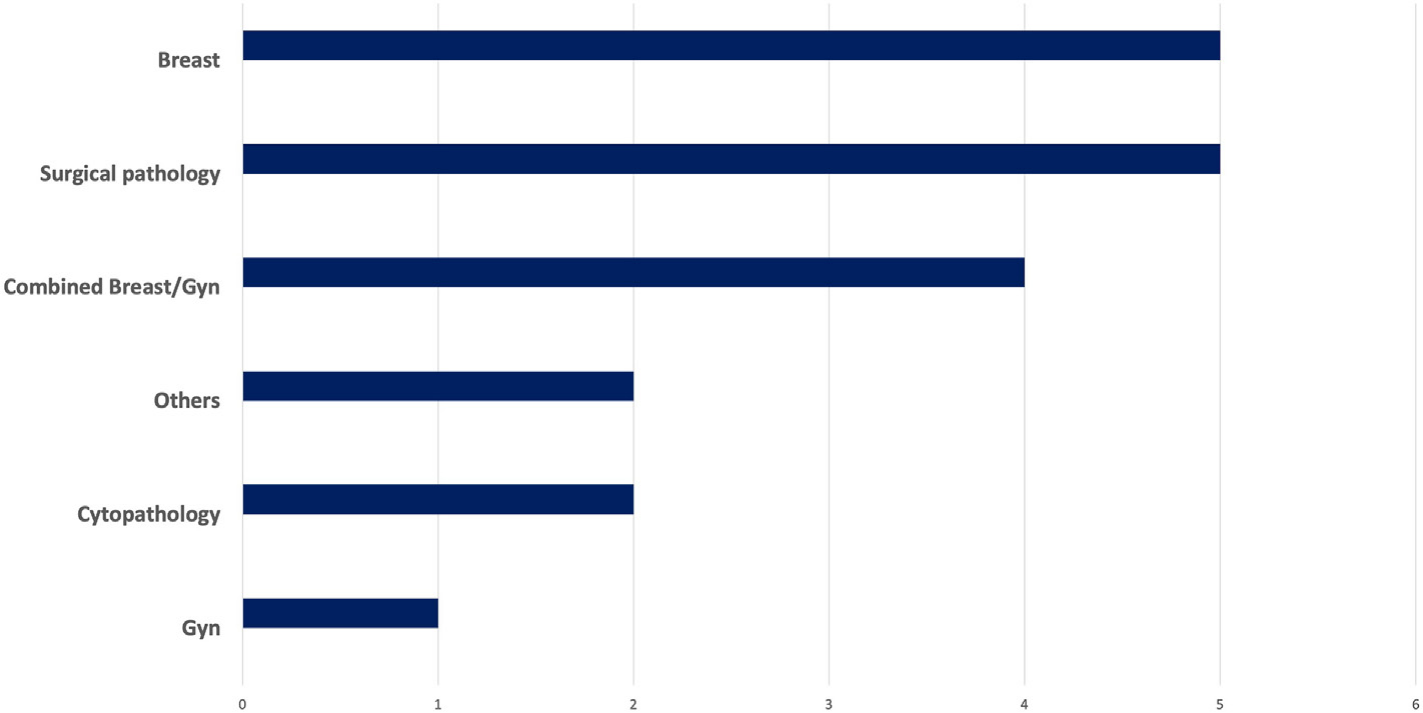
Program director's post residency fellowship training in participating programs.

Table 3 lists the majority of the self-reported information by the program directors in regard to the structure of their programs in response to the survey. Fig. 4 summarizes the average annual number of surgical pathology, cytology, intraoperative consultation (IOC), breast and gyn cases in the departments of pathology housing the combined breast/gyn fellowship training. Fig. 5 illustrates the post-residency training of the program directors of the responding programs.

**Table 3:** Characteristics of the combined breast/gyn fellowship training programs in participating institutions (n = 11).

Variable	# of programs, (%)
State license required for the fellows rowhead	
Full	5 (45.5)
Temporary	5 (45.5)
Not required	1 (9)
Research project for graduation	
Required	6 (55)
Not required	5 (45)
Fellows' sign-out cases independently	
Yes	3 (27)
No	8 (73)
Educational curriculum present	
Yes	6 (55)
No	5 (45)
Fellowship common application (2022)^a^	
Accept	16/25 (64 %)
Do not accept	9/25 (36 %)
Graduates' job destination	
Private practice	5 (46)
Academic	3 (27)
Others	2 (18)
Another fellowship	1 (9)

Other variables	Mean (range)
Breast and gyn referral/consult cases received per year.	500 (50–4000)
Number of interviews conducted for one position.	4 (1–6)
Number of practicing pathologists in the department	
Breast	5 (1->10)
Gyn	5 (1->10)
Cytology	8 (4->10)

a Information obtained from reference # 2 for all combined breast/gyn fellowship training in the US in 2022.

## Discussion

According to an American Society of Clinical Pathology (ASCP) survey conducted in 2020, fellowship training is planned by 97 % of the residents with about half of the residents plan to complete one fellowship, and 44 % reporting interest in two or more fellowships post residency.^2^ Determining the formal goals and objectives of training helps to structure the training program as well as assess the progress of trainees during the year. Table 4 describes one possible example of program goals.

**Table 4:** Goals and objectives of training in the combined breast/gyn rotation.

Patient Care
• To develop proficiency in the grossing and microscopic diagnosis of gynecologic and breast diseases.
Medical Knowledge
• To understand the concept of diseases and correlation with morphology.
• To understand the basis and clinical applications of immunohistochemistry in the discipline of gynecologic and breast pathology.
• To correlate the morphology with the clinical and radiologic presentation.
Interpersonal and communication skills
• To develop proficiency in presentation of findings to the trainees, and clinicians.
• To communicate effectively with the clinicians.
• To prepare accurate, written pathologic reports including synoptic templates.
Professionalism
• To demonstrate respect, compassion, and integrity when encountering patients, clinicians, other trainees, and staff.
• To complete tasks efficiently in a timely fashion.
• To work effectively and respectively as a team with technical and administrative staff
• To participate effectively in multidisciplinary conferences that include presentations and multidisciplinary conferences.
Systems-Based Practice
• To understand the role of quality assurance in diagnostic surgical pathology by participating in quality assurance project(s).
• To select appropriate, special studies.
• To apply current and up-to-date tumor staging and grading systems.
Practice-Based Learning
• To use case-based learning as a tool for understanding pathogenesis.
• To locate and appraise pertinent evidence from scientific studies.

Recruitment of candidates to the fellowship training is an integral part of program success. A recent survey by the Surgical Pathology and Surgical Pathology Subspecialties Fellowship Recruitment team found that 126/155 (81.3 %) of the programs support a unified approach with either full or partial participation to fill their positions. Among selective pathology programs, 60/76 (78.9 %) supported a unified approach.^2^ Eighty-two percent of the participating programs in our report post their fellowship positions on the Pathology Outlines, and 69.6 % accept College of American Pathologists (CAP) common application. The preferred method of conducting interviews is virtually, using online platforms.

Eleven out of twenty-five (44 %) of the combined breast/gyn fellowship programs are accredited while 14 (56 %) are not accredited by the ACGME. Depending on the availability of each institution, a few weeks of rotation in the clinical departments, cytogenetics and/or molecular laboratory with an emphasis on applied technology (e.g., FISH) will assist in preparing the fellow for independent practice. Our report indicates that there is no consistency among the programs in rotations for other subspecialties, with few programs offer rotations in radiology, cytogenetics, molecular, and neonatal/placental pathology. We identified that some programs require the completion of a research project for graduation, while most require scholarly activities. This suggests that there is a strong emphasis on research in all programs.

To the best of our knowledge, our study represents the first survey of the combined breast/gyn fellowship training programs. Our survey provides a unique understanding of the infrastructure opted for by the breast/gyn fellowship programs for the pathologists in training. By obtaining the perspective of the program directors, we have attempted to impart a holistic view of training in the breast/gyn subspecialty.

Limitations of our study include the low survey response rate (11/25), which limits the ability to understand the landscape. Moreover, the responding programs could be a non-representative subset due to respondent bias. The search strategy did not guarantee that all programs would be identified. We did not receive information about any discontinued programs. The information was self-reported. We contacted only program directors and did not have a means to contact program coordinators or other respondents.

In conclusion, our study provides valuable insights into the current state of combined breast/gyn pathology fellowship training. It is our hope that the information we gathered will be helpful to current and prospective trainees, as well as program leaders and other stakeholders.

## Funding disclosures

None. This research did not receive any specific grant from funding agencies in the public, commercial, or non-for-profit sectors.

## Disclosure/conflict of interest

Dr. Rohit Bhargava serves as consultant to Agilent, ImmunoGen, and GE Healthcare/Cogora. All other authors have no conflict of interest.

## Declaration of competing interest

The authors declare that they have no known competing financial interests or personal relationships that could have appeared to influence the work reported in this paper.
