# Supplementary material for: Selective breast/gynecologic pathology fellowship training in the United States: Experience of program directors
Source: Acad Pathol. 2024 Feb 14;11(1):100103. doi: 10.1016/j.acpath.2023.100103 (PMC10877681; doi:10.1016/j.acpath.2023.100103)
Supplement: Multimedia component 1 [file mmc1.docx]

**Supplemental Material 1: Survey Questionnaire.**

| 1. What is the program structure? (Academic, cancer institute …etc) |
| --- |
| 1. What state is the program located in? |
| 1. Is the fellowship ACGME accredited? |
| 1. What is the year the program was established? |
| 1. What are other fellowship programs offered by the department? |
| 1. What are the program director certification and postgraduate training? |
| 1. How many full time faculty members/practicing pathologists are in the department? |
| 1. What is a total accession, including outside referral cases in the department? |
| 1. What is the yearly average total number of intraoperative consultations for the department? |
| 1. What is the number of fellowship-related intraoperative consultation cases in a year? |
| 1. What is the average number of breast and Gyn accessions (not including GYN cytology)? |
| 1. What is the average number of breast/Gyn professional consultations? |
| 1. Is there a fixed formal curriculum or list of topics/consensus fellows must learn? |
| 1. Is prior fellowship training required for the combined breasts/kind training? |
| 1. How long is the fellowship? |
| 1. In the most recent five years, what is the most common destiny of the graduates? |
| 1. How many weeks do fellows rotate in Breast and Gyn services? |
| 1. What kind of state medical lessons are required to enroll in the program? |
| 1. Do fellows involve in teaching medical students/residents? |
| 1. Are the fellows able to sign out cases independently? |
| 1. Is completion of a research project and/or publication required for graduation? |
| 1. How many journal club presentations required by the fellows? |
| 1. How are the interviews conducted for the position? (virtual vs on site) |
| 1. How many interviews do you conduct on average for one position? |
| 1. Where the fellowship positions advertised? |
| 1. What are the elective and core rotations during the fellowship training? |
